# It's late, but not too late to transform health systems: a global digital citizen science observatory for local solutions to global problems

**DOI:** 10.3389/fdgth.2024.1399992

**Published:** 2024-11-27

**Authors:** Tarun Reddy Katapally

**Affiliations:** ^1^ DEPtH Lab, School of Health Studies, Faculty of Health Sciences, Western University, London, ON, Canada; ^2^ Faculty of Health Sciences, Hirabai Cowasji Jehangir Medical Research Institute, Pune, India; ^3^ Department of Epidemiology and Biostatistics, Schulich School of Medicine and Dentistry, Western University, London, ON, Canada; ^4^ Children’s Health Research Institute, Lawson Health Research Institute, London, ON, Canada

**Keywords:** big data, citizen science, cloud computing, data sovereignty, digital health, health systems transformation, digital platforms, mHealth

## Abstract

A key challenge in monitoring, managing, and mitigating global health crises is the need to coordinate clinical decision-making with systems outside of healthcare. In the 21st century, human engagement with Internet-connected ubiquitous devices generates an enormous amount of big data, which can be used to address complex, intersectoral problems via participatory epidemiology and mHealth approaches that can be operationalized with digital citizen science. These big data – which traditionally exist outside of health systems – are underutilized even though their usage can have significant implications for prediction and prevention of communicable and non-communicable diseases. To address critical challenges and gaps in big data utilization across sectors, a Digital Citizen Science Observatory (DiScO) is being developed by the Digital Epidemiology and Population Health Laboratory by scaling up existing digital health infrastructure. DiScO's development is informed by the Smart Framework, which leverages ubiquitous devices for ethical surveillance. The Observatory will be operationalized by implementing a rapidly adaptable, replicable, and scalable progressive web application that repurposes jurisdiction-specific cloud infrastructure to address crises across jurisdictions. The Observatory is designed to be highly adaptable for both rapid data collection as well as rapid responses to emerging and existing crises. Data sovereignty and decentralization of technology are core aspects of the observatory, where citizens can own the data they generate, and researchers and decision-makers can re-purpose digital health infrastructure. The ultimate aim of DiScO is to transform health systems by breaking existing jurisdictional silos in addressing global health crises.

## Introduction

The world is in a state of polycrisis, with multiple global crises interacting and overlapping to exacerbate the effect of a single crisis (1, 2). Issues including war, international security, and the increasing threat posed by climate change has countries across the low-, middle-, and high-income spectrum experiencing severe heat waves, cyclones, and droughts, which are exacerbating existing health and social inequities, especially among vulnerable and low-income groups (1). Managing these existential polycrisis requires systems transformation by co-ordinating decision-making across sectors to include key aspects of survival and societal functioning, such as food security (2), maintenance of law and order (3), and social support to vulnerable groups (4). However, developing and implementing systems-wide policies is extremely challenging without timely collection, analysis, and visualization of data, where disparate information from multiple sources can result in disjoint decision-making (5). From a citizen or patient perspective, the increasing burden on healthcare infrastructure, lack of resources, and financial constraints continues to prevent vulnerable populations from effectively managing or preventing existing diseases – further widening existing health inequities in particular (6).

Transformation of health systems by ethically leveraging citizen-driven big data is a critical component in overcoming these challenges to decision-making at both ends of the spectrum (policy maker to citizens) (5, 7). In 2018, the World Health Assembly passed a “Digital Health Resolution” recognizing the role of digital technologies in improving access, availability, use, and effectiveness of healthcare service delivery (7). Even though non-communicable disease risk was noted as a critical target area given the potential for addressing health behaviours (i.e., lifestyle factors such as diet), digital health interventions have depicted their transformative potential during the most-recent communicable disease crisis we faced – the Coronavirus disease (COVID-19) pandemic (8).

Digital health interventions have been used to track and manage both communicable and non-communicable disease risks by improving accessibility, service delivery, cost-effectiveness, and improved clinician capacity (6–9). However, the complexity of public health crises in the current age of polycrisis requires dynamic solutions which can be adapted to the evolving nature of individual and contextual (i.e., social, ecological, economic, political) risk factors. Digital health platforms that are powered by citizen-driven big data from ubiquitous tools can not only provide citizens with real-time support to improve their own decision-making, but also ethically relay citizen data to decision-makers for rapid responses (7, 10). These platforms can transform public health approaches to both communicable, and non-communicable management (7, 11) and potentially enable direct patient-clinician communication to mitigate and manage existing and emerging public health crises – a paradigm-changing approach that is inverting innovation by prioritizing community needs, i.e., advancing digital health for equity (10).

However, a key challenge in monitoring, managing, and mitigating public health crises is the need to coordinate clinical decision-making with systems outside of health (i.e., food systems, social welfare) (12). In this digital age, human engagement with and through ubiquitous Internet-connected devices generates an enormous amount of big data which can be used to address complex, intersectoral problems (13), and the ever-increasing usage of information technology in all spheres of human life continues to add to this growing phenomenon (14). However, historically, these big data – which traditionally exist outside of health systems – are underutilized even though their usage can have significant implications for prediction and prevention of communicable and non-communicable diseases (7).

In an attempt to address significant challenges in the usage of citizen-driven big data, and gaps in coordination of evidence-based decision-making, a Digital Citizen Science Observatory (DiScO) is being developed by the Digital Epidemiology and Population Health Laboratory (DEPtH Lab) (15) by scaling up and re-purposing existing digital health infrastructure. Although citizen science is primarily considered a research approach that is participatory in nature and can range from contribution and collaboration with citizens to co-creation of knowledge (2, 7, 10), it is rarely used in the development and implementation of digital health platforms. It is important to note that the pathway to this digital citizen science approach has been laid by decades of work in the areas of infodemiology (16, 17) participatory epidemiology (18–20), and public eHealth (18–20), where data are sourced from citizens' engagement with the internet, direct researcher engagement with citizens via internet connected devices, and patient engagement with electronic health records (21, 22).

The overlapping aspect of these foundational areas of research in digital health is the data sourced from citizens, which the proposed digital citizen science approach can advance by providing value perspective to both citizens and policy makers in enabling decisions in near real-time by inextricably linking citizen-driven big data with decision-maker rapid responses – a key gap in current health systems. Thus, the primary goal of DiScO is to transform health systems by ethically leveraging citizen-driven big data that can inform both policy maker and citizen/patient decision-making in predicting and preventing public health crises across jurisdictions.

## Leveraging existing digital infrastructure

The approach to building DiScO is to scale-up/down, replicate or re-purpose existing digital health infrastructure, as relevant, to prioritize and promote open science (23) and rapid response structures irrespective of location of development and implementation. This approach is validated by existing and expanding digital infrastructure in every aspect of society (24, 25).

Following this replicability-focused approach, DiScO is currently engaging in first step of the development process, which involves leveraging existing digital health infrastructure developed by the DEPtH Lab. This includes a digital platform which serves as a public health advisor, and has been implemented via a novel progressive web application (PWA) that can be modified to provide jurisdiction-specific public health advice (10). The PWA is linked to digital health dashboard through the “hypertext transfer protocol” (HTTP) request and “hypertext processor” (PHP) coding, an open-source server-side scripting programming language, which executes programming instructions at runtime (26). Two virtual private cloud environments, which are hosted on Amazon Web Service, securely store and communicate data between the database, PWA, and the digital health dashboard (27). The development process resulted in a replicable and scalable jurisdiction-specific digital health platform that was tested within the DEPtH Lab to confirm not only the real-time linkage between the PWA and the digital health dashboard, but also the functionality of the PWA in its ability to provide citizens with real-time public health advice, while relaying aggregated and anonymized big data to the digital health dashboard to enable jurisdictional decision-making.

**Figure 1:**
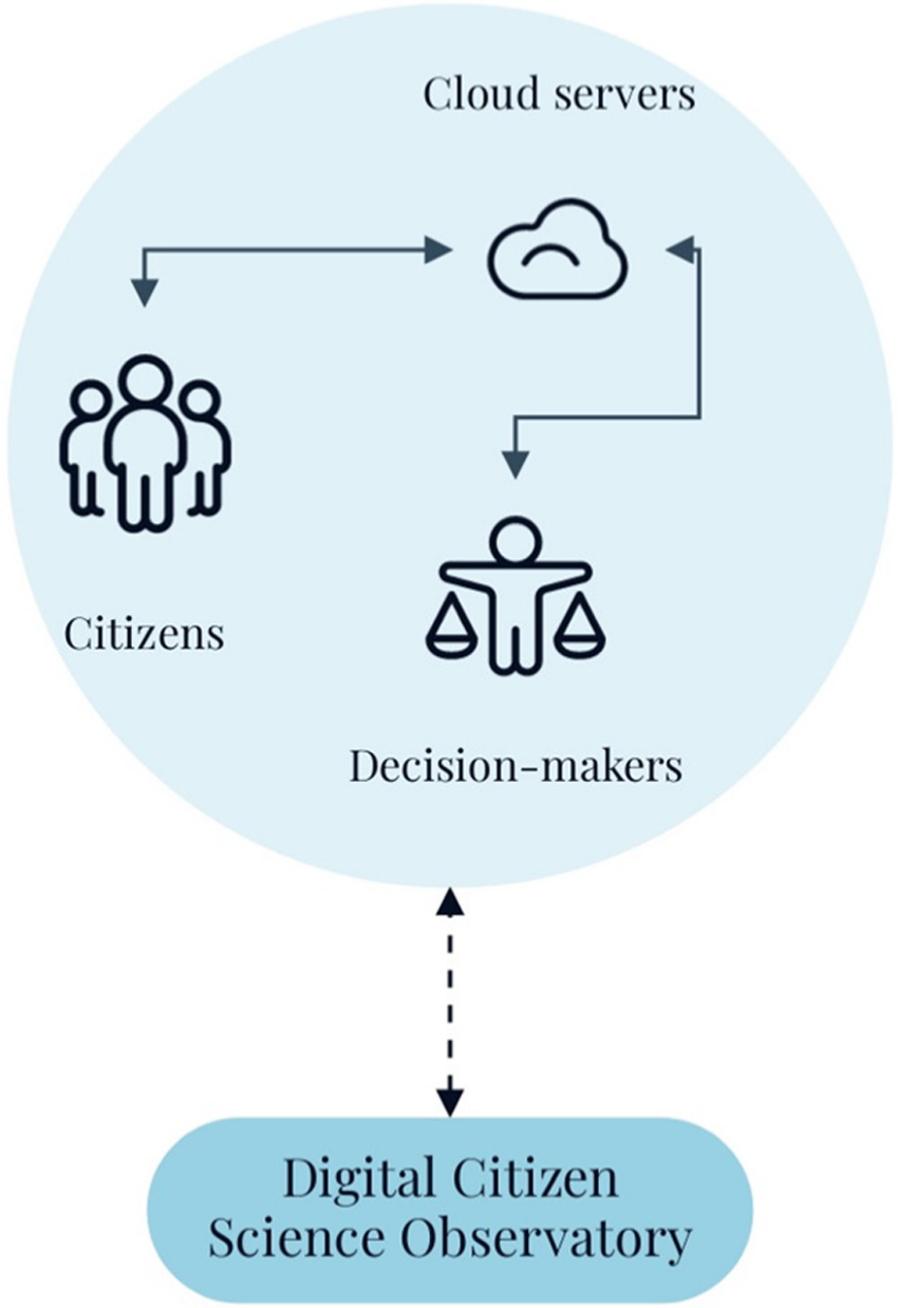
Basic DiScO structure.

**Figure 2:**
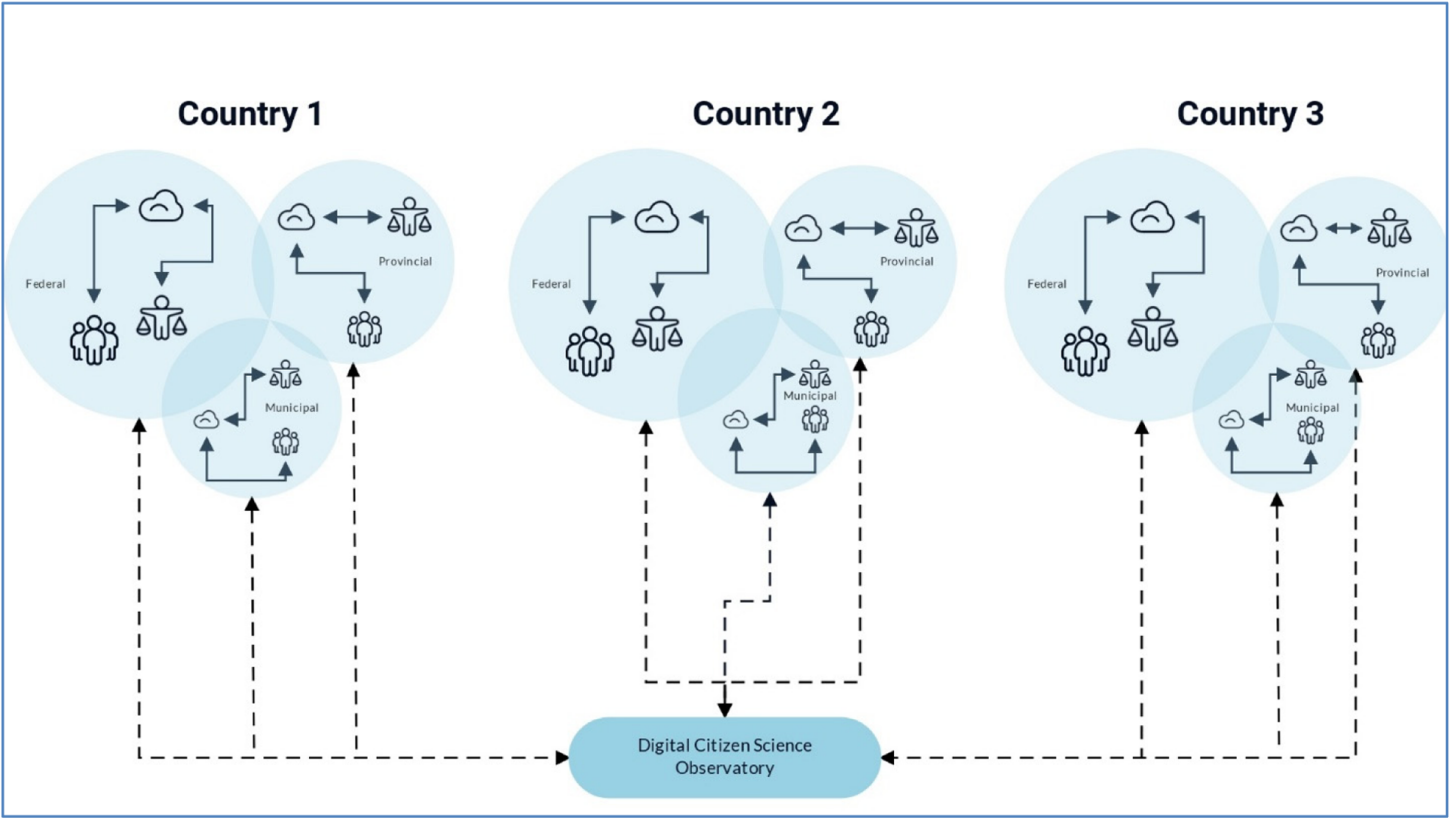
Scaled-up and scaled-down versions of DiScO.

In essence, this existing digital health infrastructure provides a “value perspective” to citizens, i.e., the big data they contribute is used for their personal and larger community's benefit, and ultimately can be used to inform evidence-based decision-making through rapid-responses in real-time. The value perspective, which results in public health advice, is critical for the success of the infrastructure scale-up because it motivates the citizens to participate in the digital health platform – a core need for the operationalization digital citizen science. Although citizen science is primarily considered a research approach that is participatory in nature and can range from contribution and collaboration with citizens to co-creation of knowledge (2, 7, 10), it is rarely used in the development and implementation of digital health platforms. This approach to citizen science for the development of digital health dashboards enables new opportunities, such as scaling them up to implement DiScO across jurisdictions (Figures 1, 2) to address public health crises from a systems perspective, i.e., an observatory that goes beyond “observation” and enables implementation of interventions.

The solid arrows depicting data flow within jurisdictions highlight decentralized and jurisdiction-specific collection and storage of big data, as well as implementation of rapid responses to mitigate emerging crises, while utilizing standardized and scalable digital infrastructure - depicted by dotted arrows going from the observatory to individual jurisdictions. The dotted arrows going from individual jurisdictions to the Observatory portray data sovereignty of jurisdictions, where only completely de-identified, and irreversibly anonymized data can be centrally shared to facilitate global solutions, while abiding by individual jurisdictional data regulations.

This implementation using digital citizen science can potentially improve uptake from citizens, as well as enhance scalability and adaptability across jurisdictions. Thus, it is a digital system that is not only developed to address needs of the citizens, but also to meet decision-maker goals in addressing citizen needs (28). An Observatory with this approach can potentially transform health systems across jurisdictions (29), while promoting health equity by prioritizing and addressing the needs of citizens in near real-time (30, 31). Most importantly, the same digital health infrastructure could be modified to address jurisdictional-specific issues ranging from climate change to infectious diseases (7, 10) and non-communicable diseases (32) to systemic issues such as food security (11) – an approach that currently lies beyond the scope of health systems.

## Implementation framework

The methodology informing the development and implementation of DiScO intersects digital citizen science, digital epidemiology, and data science to directly engage with citizens and ethically leverage big data that can not only be securely stored in cloud servers, but also relayed to decision-makers in near real-time for rapid decision-making. To implement this methodology, the observatory utilizes the Smart Framework (32), which integrates citizen science, community-based participatory research, and systems science through ubiquitous tools to conduct population health interventions in the digital age (10).

The digital health revolution has transformed the collection and analysis of electronic health records, as well as physiological and behavioral measurements at the individual level. However, our health care systems are primed for benefiting downstream service providers for disease management rather than promoting upstream policies to prevent disease development (7). Another concern is the lack of ethical engagement in digital health, where the power resides predominantly with researchers and providers.

DiScO can potentially play a role in the transformation of the health systems approach to citizen/patient engagement by: (1) Implementation of ethical real time surveillance to assess community health by subjective and objective (sensor-based) longitudinal data gathering; (2) Implementation of near real time integrated knowledge based on the data provided by crowdsourced citizens to increase awareness of health-promoting practices; (3) Implementation of evidence-based real time communication that not only link citizens to health care services, but also ensure real-time support using digital health dashboards. (4) Development of decision-making dashboards at the back end, which enhance real time information sharing and data analytics that would inform community decision making to mitigate risk and ensure productivity during health crises; (5) Development of precision prediction models to mitigate risk of existing and emerging public health crises (7, 10).

Establishing such an observatory requires operationalizing its theoretical underpinnings in order to provide stakeholders with a clear pathway to decision making, which is provided by the Smart Framework via an approach which ethically repurposes citizen-owned ubiquitous communication devices (29). Smartphones, in particular, have revolutionized the ability to sense, share, and link big data (29). This potential for repurposing ubiquitous devices has magnified since the COVID-19 crisis because smartphones have the reach to enable equitable information and service access. Smartphone-based apps therefore have the capacity to source big data to inform policies through the voice of the citizens themselves (7).

## Discussion

The flexibility of digital platforms for scale-up/down and replicability opens up enormous opportunities to operationalize DiScO for health systems transformations across jurisdictions – whether within a single country or across multiple nations (Figure 2). For instance, from a Canadian health systems perspective, health care implementation is the mandate of provinces and territories as there is no federal health care system (33, 34). Thus, the natural authorities to introduce and implement digital health platforms would be the provinces and territories who receive resources from federal healthcare budgets. Federal government in such a system can play a larger role in mandating such platforms, which will eventually play a part in enrolling entire populations – a critical factor in the success of digital citizen science platforms.

However, digital citizen science platforms can exist outside of traditional health care systems, as they are able to ethically leverage big data from multiple systems – an approach that has its own advantages of systems integration (11, 35). Because digital citizen science platforms can exist outside of health systems, they do not have to be limited to government ecosystems. In fact, digital citizen science platforms have formerly been used for non-communicable disease monitoring and interventions, which are implemented outside of health systems (36).

Irrespective of where DiScO is housed, whether integrated with existing health systems or enabling it to function independently outside health systems depending on jurisdictional needs, there are obvious benefits to the health of populations given its capability to develop precision prediction models that can respond to citizen needs in near-real-time by using their own ethically obtained data (10). Beyond the obvious population health impacts, operationalization of DiScO has social and societal benefits, including potential community empowerment through citizen science approaches, where citizens are not only brought closer to traditional decision-making processes by sharing their own big data, but can also play a role in countering digital misinformation (7). This assertion is based on the potential that citizens will trust the evidence that is generated by using their own data, especially if these data are used to make decisions by policy-makers – decisions that have implicit impact on the lives of the citizens. Moreover, the observatory's structure is set up to provide real-time support to citizens – support that is informed by aggregating their own data, a key approach that can minimize misinformation and enable better decision-making at the household level.

In terms of decision-maker benefits beyond the use of big data for rapid decision-making, development of jurisdiction-specific decision-making dashboards can transform decision-maker approach to managing existing and emerging crises (10). Ultimately, in a highly connected and complex world, there is a need for health systems transformation which requires thinking beyond health systems by taking systems thinking approach, i.e., systems integration (37–40).

Operationalization of DiScO has the potential to address the varied health and social impacts of polycrisis that go beyond current scope of health systems, such as rapid responses for climate change mitigation and adaptation, which inadvertently put enormous stress on current health care resources (41, 42). The increasing threat of pandemics such as COVID-19 (5, 12, 43) demonstrate the limitations of health care systems in addressing complex and overlapping crises. The pandemic experience forces us to think beyond traditional operation of health systems and move towards transformation that enables systems integration, which can be enabled by leveraging big data ethically directly from citizens (10).

Paradoxically, the ability to address these polycrisis using digital transformative approaches, comes with its own risk of data safety, security, and sovereignty. Thus, protecting privacy and anonymity of citizens through strong encryption processes will be the highest priority (44, 45). Moreover, before any citizen engagement, obtaining informed consent must be mandatory because, from an academic ethics approval perspective, it is not possible to obtain data from citizens without obtaining informed consent. While informed consent does pose challenges in obtaining big data (46), it is imperative that informed consent remain central to enable digital data collection, irrespective of the type of data being sourced (47, 48) – an ethical approach that can be solidified by digital citizen science, where citizens have the power to not only share data after providing informed consent, but also have the technological option to manage data sharing (31). Thus, going above and beyond traditional ethics protocols, citizens participating in the Observatory would be provided the option to dropout and/or delete their own data (i.e., embedding of open-source features). However, to truly conduct ethical surveillance, data ownership is essential, where citizens are able to participate in data visualization, analysis, and knowledge translation (29, 32, 49, 50). Ethical citizen engagement will be paramount to not only avoid invasive surveillance (51), but also to enable equitable engagement irrespective of the geographic region of the citizens. This approach not only complies with jurisdictional data legislation by integrating advanced encryption processes and secure cloud computing, but also advances ethical big data collection through decentralization of technology and data sovereignty of citizens, i.e., social innovation in health systems transformation.

Perhaps more proximal to current health systems, integrating DiScO with Electronic Health Records (EHRs) could transform healthcare by enriching clinical data with contextual, community-generated insights, aligning with initiatives like the European X-eHealth project (52), which promotes real-time and cross-border data exchange to support personalized care. DiScO's data on health determinants, such as air pollution, could be directly incorporated into EHRs, allowing providers to evaluate broader environmental and social triggers affecting patient health. For instance, real-time data integration could enable EHR-based alerts or decision-support tools to guide proactive interventions for at-risk individuals, supporting a more preventive approach to care (53). With real-time insights, clinicians could implement adaptive care measures, recommending preventive actions, medication adjustments, or follow-ups during pollution peaks (54). This integration also aligns with studies on patient-generated health data, which show that incorporating real-world health experiences can improve chronic disease management (55). Evidence suggests that adding social determinants of health information to EHRs is generally acceptable to patients, as it makes health data more representative and personalized (56). Research has also demonstrated the potential for EHR-based real-time geocoding and external data linkages to advance precision medicine, as seen in approaches to Type 2 diabetes prevention where environmental exposures inform individualized care (57). This holistic model could facilitate more sustainable, responsive healthcare systems that incorporate both clinical and community data across jurisdictions.

Overall, this approach to social innovation, which ethically leverages the power of digital technology is centred on decentralization and democratization of technology, as well as data sovereignty of each participating citizen irrespective of jurisdiction. The decentralization is three-tiered – (1) Jurisdiction-specific collection and storage of big data, as well as implementation of rapid responses to mitigate emerging crises, while utilizing standardized and scalable digital infrastructure; (2) The development and implementation of PWAs, which takes the platform ecosystem away from the monopolization of big technology launch platforms, i.e., Google Play Store and Apple Store. (3) The backend dashboards will be set up to decentralize access to information, hence enhancing direct citizen data ownership and access, yet enabling usage by decision-makers within, and outside health systems i.e., democratization of technology. The decentralization and democratization of technology, and data sovereignty of citizens are the foundation for secure health systems transformation. Ultimately, this multi-tiered strategy of simultaneously involving and protecting citizens in big data collection translates to a scenario of high reward for high risks that build on population health, social, societal, and decision-maker benefits.

### Addressing jurisdictional challenges in privacy, regulation, and infrastructure

For DiScO to operate effectively across diverse international jurisdictions, there are essential considerations regarding data privacy, variations in data protection regulations, and evolving digital infrastructure capabilities. Despite robust encryption and informed consent practices, managing data security across borders requires stringent protocols and alignment with varying national and international data protection standards (58). DiScO will adopt a multi-layered encryption model that adapts to the specific regulatory requirements of each region while aligning with overarching data protection laws, such as the General Data Protection Regulation (GDPR) in Europe (59), the Health Insurance Portability and Accountability Act (HIPAA) in the United States (60), and similar frameworks worldwide. The observatory will store data within regional cloud environments to facilitate local compliance and conduct regular audits and updates to address new privacy challenges as they emerge.

Given the sensitive nature of health data, regulatory oversight is key to enabling public trust and ensuring ethical data use. Collaborations with government bodies and independent regulatory authorities will be established to create transparent and localized data governance protocols. Working with these entities can align DiScO's operations with local legal frameworks while providing citizens with clear, accessible information on how their data are collected, stored, and used. Such partnerships will also support jurisdiction-specific oversight, with governments and regional bodies helping to enforce data use policies and mitigate any potential misuse.

The observatory's goal to operate across jurisdictions with varying levels of digital infrastructure requires adaptable technology and flexible operational frameworks. DiScO's modular, cloud-based architecture will accommodate regions with differing digital capacities, allowing the platform to scale up or down as needed (58). Additionally, DiScO will adopt internationally recognized standards for data interoperability, such as those established by the International Organization for Standardization (ISO) (61), enabling seamless data exchanges while adapting to local infrastructure constraints. This approach allows for a unified yet flexible data-sharing system that can address evolving public health issues across borders.

Nevertheless, the differences in regulatory environments across the world introduce a layer of complexity that must be navigated carefully. DiScO will create a regulatory compliance strategy that maps major international and local regulations to provide a comprehensive guide for maintaining alignment across jurisdictions. However, development and implementation of DiScO on a global scale requires comprehensive global stakeholder input to understand and account for the complexity of implementing digital health infrastructure across international jurisdictions (58).

The DEPtH Lab recently conducted a systems mapping study – an approach for obtaining rapid stakeholder input, particularly to capture systems thinking (58). This systems mapping study, which involved international decision-makers and stakeholders representing eight countries, resulted in generating key action items, which included advancement of digital literacy and internet equity across jurisdictions – two key drivers for the success of digital transformation of health systems (62, 63). Moreover, systems mapping also identified that capacity building of researchers, decision-makers, and citizens themselves is necessary for effective digital transformation of health systems. Ultimately, digital transformation of health systems will depend on citizens' capacity and motivation to share their non-health systems data consistently, and consent data linkages with their personal EHRs. To achieve this, DiScO will enable exploration of the potential and capacity of digital citizen science to source and link big data both within and outside health systems to facilitate digital transformation of health systems across international jurisdictions.

## Conclusion

Health systems transformation in the age of polycrisis requires coordination of clinical decision-making with systems outside of health (i.e., food systems, social welfare). In the 21st century, human engagement with and through Internet-connected ubiquitous devices generates an enormous amount of big data, which can be used to address complex, intersectoral problems. In an attempt, to address significant challenges associated with the use of citizen-driven big data, as well as existing gaps in coordination of evidence-based decision-making, DiScO is being developed to ethically leverage citizen-driven big data develop local solutions for global problems. The benefits of the Observatory go beyond obvious promotion of population health, to include social and societal advancements, and cutting-edge decision-maker approaches to solving complex problems. The success of the Observatory will ultimately depend on balancing high risks with high rewards while replicating and implementing the technology across jurisdictions.

## Data Availability

The original contributions presented in the study are included in the article/Supplementary Material, further inquiries can be directed to the corresponding author.
